# Optimizing the Antimicrobial, Antioxidant, and Cytotoxic Properties of Silver Nanoparticles Synthesized from *Elephantorrhiza elephantina* (Burch.) Extracts: A Comprehensive Study

**DOI:** 10.3390/plants14050822

**Published:** 2025-03-06

**Authors:** Matshoene V. Motene, Charity Maepa, Muendi T. Sigidi

**Affiliations:** 1 Department of Biochemistry and Microbiology, Faculty of Sciences, Agriculture and Engineering, University of Venda, Private Bag X5050, Thohoyandou 0950, South Africa; matshoenev@gmail.com; 2 Laboratory for Microscopy and Microanalysis, Faculty of Natural and Agricultural Sciences, Private Bag X20, Hatfield 0028, South Africa; charity.maepa@up.ac.za

**Keywords:** *Elephantorrhiza elephantina*, silver nanoparticles, cytotoxicity, green synthesis, UV–VIS, TEM

## Abstract

The green synthesis of silver nanoparticles (AgNPs) using *Elephantorrhiza elephantina* (Burch) bulb extracts and evaluation of their antimicrobial, cytotoxic, and antioxidant properties were investigated. The crude plant extracts were prepared using distilled water, ethanol, and methanol for a comparison. Silver nanoparticles were synthesized and characterized via UV–Visible spectroscopy (UV–VIS), transmission electron microscopy (TEM), and X-ray diffraction (XRD). The formation of silver nanoparticles was confirmed using the UV–VIS spectra at 550 nm. The TEM confirmed the nanoparticle morphology as a mixed dispersed sphere, oval, and triangular shapes with a size range of 7.8 nm to 31.3 nm. The secondary metabolites were detected using TLC, DPPH, and LC-MS. Antimicrobial activity was assessed based on agar-well diffusion; cytotoxicity was examined through MTS assays. Various phytochemical constituents were detected through TLC and LC-MS. The crude extracts and methanol-extract-capped AgNP were able to scavenge free radicals, as shown by the developments of inhibitory bands on the TLC plate. The agar well diffusion test revealed that the AgNP capped methanol extract had potent antimicrobial activity against Gram-positive and Gram-negative multidrug resistant bacteria in comparison with penicillin and neomycin, with inhibition zones ranging between 10 mm and 14 mm for the methanol-extract-capped AgNP. The in vitro MTS assay revealed that methanol crude extracts and methanol-extract-capped AgNP had a less cytotoxic effect on the HEK293 cells in comparison with untreated cells (control). We therefore conclude that methanol was the best reducing solvent with the best overall nanoparticle morphology and performance in antimicrobial and cytotoxicity, in comparison to ethanol and distilled water.

## 1. Introduction

Previously, humans especially in rural-based communities, had to rely on drugs from nature (plants, fungi, and animals) as worldwide, medicinal systems, which had been developed thousands of years ago, relied heavily on herbal medicine. There are extensive documented records of plants used in medicinal practices, in various parts of the world, like the traditional Chinese medicine Kampo, Ayurvedic, and those from Europe, Africa, Australia, and the Americas [1]. Medicinal plants have been used to treat a diversity of diseases and infections, from acute to chronic cases, and for nutritional purposes [2,3,4]. The rhizome, roots, leaves, and stems of *E. elephantina* are reported to possess diverse phytochemicals.

Medicinal properties are due to the presence of various phytochemicals, like anthrocyanidins, anthraquinones, esters, fatty acids, phenolic compounds, flavonoids, glycosides, polysterols, saponins, sugars, tannins, and triterpenoids—which are used to treat or manage various human and animal ailments and diseases, throughout its distributional range in southern Africa [4,5]. A total of 42 and 14 human and animal ailments and diseases, respectively, are treated by herbal medicines prepared from *E. elephantina*. These reports are from all the countries where *E. elephantina* is indigenous, and the country with the highest ethnomedicinal uses is South Africa (45) based on 25 literature records [2].

Nanotechnology has gained much interest and has a wide range of processes that decrease toxic substances to remediate the environment. Modern alternative metal nanoparticle synthesis includes the use of inactivated plant tissue, plant extracts, exudates, and other parts of living plants [6]. The identification and use of nanoparticles was a breakthrough in medicinal studies. Currently, nanoparticles are developed for drug, heat, and light delivery to specific cells [7]. The application of nanoparticles reduces damage to healthy cells in the body, in addition to allowing for the early detection of diseases because they are engineered to be attracted to diseased cells, permitting the direct treatment of these cells. In one conducted study [8], it was observed that nanoparticles prevented the replicative function of pathogenic cells by affecting the membrane permeability and deteriorating cell growth behavior, eventually leading to cell death.

Extracts obtained from various medicinal plants have been popularly used for the reduction of metal ions to nanoparticles; this is due to their phytochemical diversity [9]. These green synthesized nanoparticles are often found to be biocompatible and more bioactive than their individual counterparts synthesized by other methods and the plant extracts used in the synthesis. An increase in bioactivities and biocompatibilities can be accredited to the compaction of phytochemicals with the nanoparticles [10].

Silver nanoparticles have been reported to have wide range of applications; they are known for their antimicrobial properties and have been used for years in the medical field for antimicrobial applications [11]. In recent years, many researchers have focused on the development of modified or novel synthetic strategies for silver nanoparticles, in contrast to the use of conventional methods, which are strongly associated with toxic environmental footprints [12]. This study reports on the antimicrobial activities of silver-capped *E. elephantina* nanoparticles against pathogenic bacteria.

Knowing the inducing effect of nanoparticles, it can be assumed that the combined effect of nanoparticles and *E. elephantina* would have potent antimicrobial activity. In this study, silver nanoparticles capped with a medicinal plant were tested for biological activity against pathogenic microorganisms and toxicity on human cells.

## 2. Materials and Methodology

### 2.1. Chemicals and Reagents

The chemicals and reagents used in the study were obtained from different sources as follows: 2-2-diphenyl-1-picrylhydrazyl (DPPH) from Sigma-Aldrich (Saint Louis, MO, USA); dimethyl sulfoxide (DMSO) and ammonium hydroxide (NH_4_OH) were purchased from Rochelle Chemicals (Steeledale, Gauteng, South Africa); ethanol and methanol were obtained from Merck Chemical (Rahway, NJ, USA); ferric chloride hexahydrate, silver nitrate (AgNO_3_), tri-sodium citrate, and hydrochloric acid were purchased from Associated Chemical Enterprise (Gauteng, South Africa).

### 2.2. Extraction

*Elephantorrhiza elephantina* bulbs were collected from Mafukani, Limpopo, South Africa; thereafter, they were thoroughly washed with distilled water to remove soil residues and potential contaminants. The roots were cut into small pieces before drying under a shade and then milled into fine powder, using an electric grinder. The plants’ extracts were macerated by soaking 50 g of it into 500 mL of distilled water (W), ethanol (E), and methanol (M) for 72 h; then the extracts were dried using a rotary evaporator (BUCHI, Flawil, Switzerland), while the water extracts were dried in a freeze-drying process (EPIC Freeze Dryer, Millrock Technology, Kinston, NY, USA).

### 2.3. Synthesis of AgNPs

The green synthesis of silver nanoparticles was performed as described by Tamilarasi and Meena [9], with slight modifications. An aqueous solution (1 mM) of silver nitrate (AgNO_3_) was prepared and used for the synthesis of Ag NPs. From the prepared crude extract, and 6 mL was added to 40 mL of a 1 mM AgNO_3_ solution for the reduction of Ag+ ions. The synthesis of silver nanoparticles was carried out at room temperature for 24 h in the dark. The silver nanoparticle solution obtained was purified by repeating centrifugation 3 times at 10,000 rpm for 10 min followed by redispersion of the pellet into acetone, and air dried.

### 2.4. Characterization of AgNPs

The synthesized nanoparticles were subjected to different characterization techniques. The UV–visible spectrophotometry (UV–VIS), Shimadzu Spectrometer (Kyoto, Kyoto, Japan) was used to obtain the spectra, operated at a resolution of 5 nm in a range of 300–800 nm. About 1 mL of the AgNPs solution was dispersed on a film and analyzed in the spectrometer. To obtain the size and shapes of the NPs, a small amount of AgNPs was dispersed in 1 mL of ethanol and sonicated for 5 min. The dispersed solution was then dropped onto a copper grid, using a micropipette. The grid was allowed to air-dry before imaging at 200 kV with the JEOL JEM-2100F Field Emission Electron Microscope (Hitachi High-Tech, Tokyo, Japan). The XRD of the AgNPs was carried out at a current of 30 mA and a voltage of 40 kV with Cu Kα1 radiations, using a D8 Rich Seifert P3000 (Hamburg, NY, USA) with Cu Kα (λ = 1.5405 Å) radiation. The silver nanoparticles were stored in 15 mL Eppendorf tubes at 4 °C for further analysis.

### 2.5. Phytochemical Analysis

The aqueous extracts of *E. elephantina* and AgNPs were subjected to a qualitative phytochemical analysis following the procedure described by Renishiya et al. [13], to screen for active components, such as alkaloids, flavonoids, saponins, terpenoids, and tannins, as well as a quantitative analysis using thin-layer chromatography (TLC) following a procedure previously described by Lekganyane et al. [14]. The thin-layer chromatography protocol allows for the separation, identification, and comparison of the chemical constituents of different plant extracts. The use of multiple solvent systems enables the detection of a wide range of compounds with varying properties. The samples were dissolved with acetone to create a stock solution with a concentration of 10 mg/mL. A 10 µL aliquot of each sample was then loaded onto an aluminum-backed TLC plate (Sigma-Aldrich, Saint Louis, MO, USA). The TLC plate was developed in three distinct solvent systems, each with varying polarity and pH levels. The solvents used were a benzene/ethanol/ammonium solution (BEA) (10:2:0), a non-polar/basic system; chloroform/ethyl acetate/formic acid (CEF) (10:8:2), an intermediate polarity/acidic system; and ethyl acetate/methanol/water (EMW) (10:1:35.1), a polar/ neutral system.

### 2.6. Antioxidant Testing

The plant extracts and AgNPs were screened for the presence of potential antioxidant compounds. The chromatograms were prepared in a similar manner to the phytochemical analysis using thin-layer chromatography (TLC). The TLC plates were sprayed with 2, 2-diphenyl-1-picrylhydrazyl (DPPH) to detect compounds that possess antioxidant activities [15].

### 2.7. LC-MS Analysis

Further phytochemical analysis was performed using an LC-QTOF-MS, model LC-MS 9030 (Shimadzu, Kyoto, Japan). The compounds were identified between 200 and 600 nm of a 4 nm step. The mobile phase of the high-performance liquid chromatography (HPLC) consisted of 0.1% (*v*/*v*) formic acid in water (solvent A) and 0.1% formic acid:acetonitrile (1:1, *v*/*v*) (solvent B). A 100 mm × 2.1 nm with a particle size of 2.7 µm C18 column (Shim Pack Velox, Shimadzu, Kyoto, Japan) was run at 26 °C, with an injection volume of 5 µL and a 1 mL/min flow rate. The compounds were categorized using liquid chromatography mass spectrometry (LC-MS) based on their absorbance spectrum, retention time, and comparation of mass fragmentation from the literature.

### 2.8. Antimicrobial Testing

The antibacterial activities of silver nanoparticles were determined using the well-diffusion method as described by Kasithevae [8]. The zones of inhibition were recorded in millimeters (mm). Briefly, bacterial suspensions were prepared with the turbidity of 0.5 McFarland Standard. Mueller–Hinton agar plates were inoculated with *E. coli*, *K. pneumoniae*, and *S. aureus*. Wells with a diameter of 6 mm were cut, using a cork borer, and filled with 30 µL of 1 mg/mL of green synthesized silver nanoparticles and 30 µL of a 1 mg/ML solution of reference samples (plant extract and chemically synthesized silver nanoparticles), while distilled water was used as a negative control. Plates were incubated for 24 h at 37 °C, after which, the growth inhibition zone diameters were measured.

### 2.9. Cytotoxicity Testing

The human embryonic kidney cells (HEK293), (AddexBio, Pimpernel, San Diego, CA, USA) were used in this assay as described in the literature by Wang et al. [16], with modifications. Cells were cultured in DMEM low glucose and RPMI with 10% fetal bovine serum and 10% penicillin–streptomycin in a 37 °C humidified incubator with 5% CO_2_. In this assay, three separate experiments (I, II, III) were conducted for reproducibility. Viable cell counts (Table 1) were created, using a TC10 Automated Cell Counter (Bio-Rad, Hercules, CA, USA).

For each experiment, 100 µL of the cells, at 1 × 10^5^ cells/mL, was seeded in each 96-well plate and incubated for 4 h to allow for adherence. In a separate 96-well plate, serial dilutions of the compounds (plant extracts and silver nanoparticles (10 mg/mL stock)) were prepared in duplicate. The first row of wells contained 300 µL of normal growth medium (NGM), and the wells below contained 150 µL of NGM. An amount of 150 µL was aliquoted into each well of the first row and mixed well and then diluted at a ratio of 11:1 until the last row of wells. After adherence of the cells, 100 µL of the compounds was transferred to the 96-well plate containing the cells and incubated for 72 h at 37 °C; this procedure was repeated 3 times. Negative standards without extract were used for a comparison with the experimental wells.

After 72 h of incubation, 20 µL of CellTiter 96^®^ Aqueous One Solution Reagent (Promega, Madison, WI, USA) was pipetted into each well of the 96-well plate containing the samples in 200 µL of the culture medium and then incubated in a humified, 5% CO_2_ atmosphere, at 37 °C for 4 h. At 2 h intervals, the plates were read, using a UV-VIS (SpectraMax^®^ ABS Plus, Molecular Devices, San Jose, CA, USA) at 490 nm. The principle of the MTS assay is based on the change of tetrazolium salt into a colored and aqueous soluble formazan product, caused by the viable cells’ mitochondrial activity at 37 °C [17].

### 2.10. Statistical Analysis

Each experiment was performed at least in triplicate. All data shown in the tables and figures were quantified using Microsoft Excel, ImageJ 1.53, and software Origin Pro 9. The mean and standard deviations were calculated.

## 3. Results and Discussion

### 3.1. Extraction Yield and Synthesis of Silver Nanoparticles

**Figure 1:**
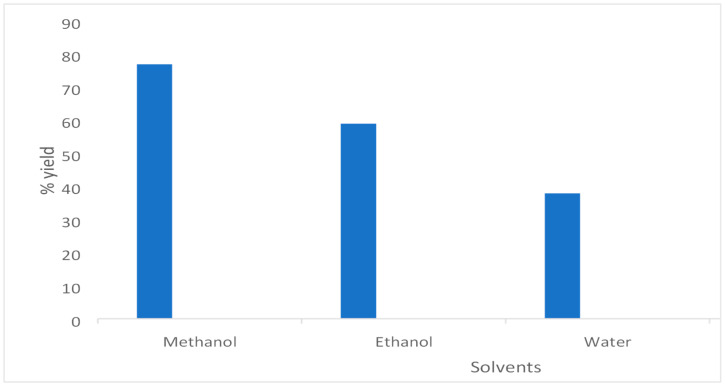
Percentage yield after extraction with methanol, ethanol, and water.

The selection of solvents in plant-based antimicrobial compound extraction is crucial for obtaining the desired bioactive elements. Solvents like water, ethanol, methanol, chloroform [5], and dichloromethane are widely used, each offering different efficacy levels and yields. The impact of the solvents on the extraction yield is evident in the comparison of 40%, 60%, and 80% extracts using water, ethanol, and methanol, respectively, as shown in Figure 1. Water, a universal solvent, yielded smaller quantities due to lower solubility for certain polar compounds. Conversely, ethanol and methanol gave better yields by dissolving a broader spectrum of compounds. Solvent polarity plays a crucial role, by extracting compounds according to their polar or non-polar nature, thus influencing the medicinal properties. Balancing the yield, safety, cost, and environmental impact is key in optimizing extraction methods. The yield was calculated using the following formula:

Y (%) = M (extracted compounds)/M (Initial plant material) × 100

**Figure 2:**
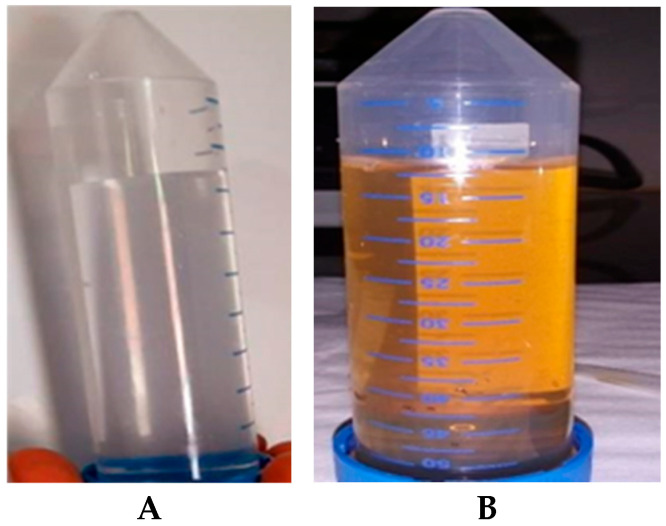
Silver nanoparticles synthesized using *E. elephantina*, after 24 h. The color transitioned from clear (**A**) to light brown (**B**), indicating the formation of silver nanoparticles.

Green synthesized AgNPs capped with *E. elephantina* were recorded based on the observed color changes of the reaction mixture from clear (Figure 2A) to brown (Figure 2B) liquids after 24 h, confirming the surface plasmon resonance (SPR) [18].

### 3.2. Characterization of AgNPs

#### 3.2.1. UV–VIS Spectrophotometry

**Figure 3:**
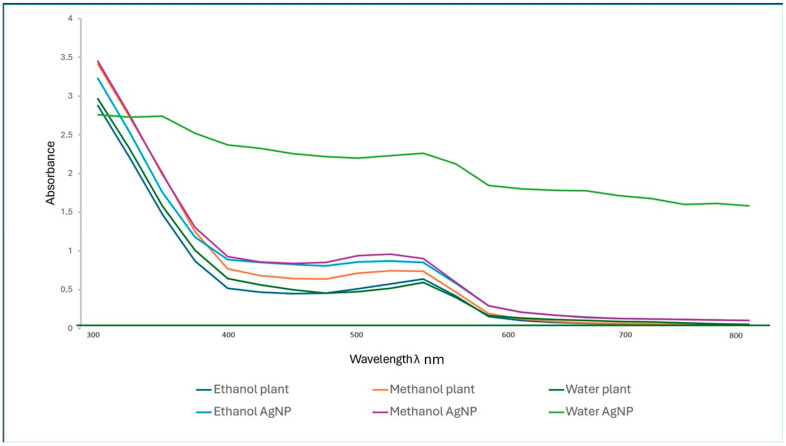
UV–VIS spectra of silver nanoparticles and *E. elephantina* extracts.

The UV–VIS absorbance was used in the characterization of the formation and stability of the final colloid. After a 24 h incubation at room temperature, the stability and formation of the nanoparticles were determined using UV–visible spectrophotometry with a 300–800 nm wavelength range. The solutions from the synthesized nanoparticles from the water, ethanol, and methanol extract showed a surface plasmon resonance (SPR) peak at 550 nm (Figure 3). The SPR occurred because of the conduction and valence bands lying close to one another in a metal nanoparticle [18]. This is because of the collective oscillation of the free electrons of silver nanoparticles in resonance with the UV–VIS light wave [19,20]. The water-extracted silver nanoparticles [AgNP W] showed a second band at 350 nm. This phenomenon was due to the formation of agglomerated or large particle sizes over time [21].

#### 3.2.2. Transmission Electron Microscopy

**Figure 4:**
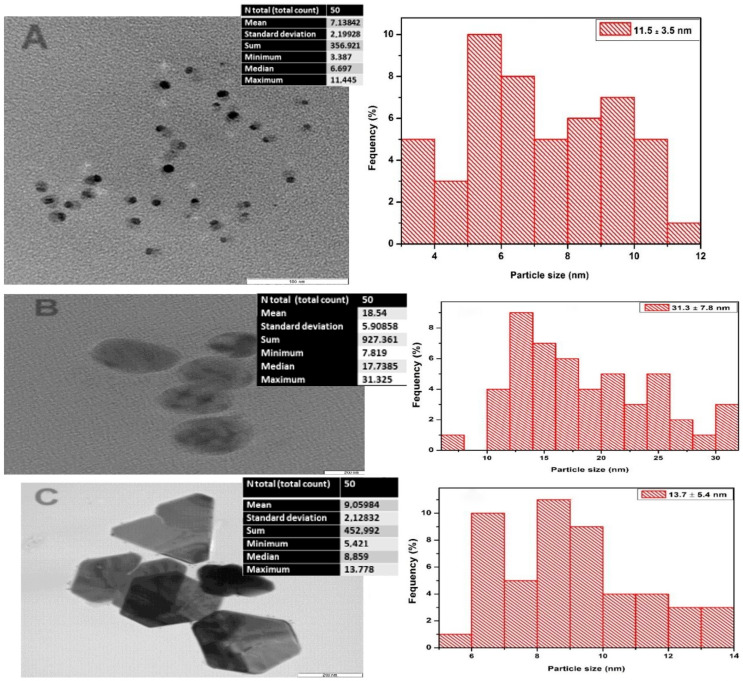
TEM images of E. elephantina-capped AgNPs extracted using different solvents, (**A**) water, (**B**) ethanol, and (**C**) methanol; and histographs showing size distributions of the nanoparticles. Different solvents produced different shapes and sizes of silver nanoparticles.

Generally, the silver nanoparticles synthesized using the *E. Elephantina* crude extracts were well dispersed, with a few agglomerated in certain areas. The average morphology of ethanol and methanol solvent extracts (or AgNPs (E and M)) were observed to be more spherical and oval in shape, while the methanol solvent extracts (or (AgNPs W)) were triangular and irregular. The average size distributions of the AgNPs were 11.5 ± 3.5 nm, 31.3 ± 7.8 nm, and 13.7 ± 5.4 nm, respectively, for water, ethanol, and methanol, as indicated by the histogram in Figure 4.

The nanoparticle morphologies were influenced by the solvents used for extraction and the phytochemical compounds present in the plant extracts, with terpenoids, flavonoids, polyphenols, and polysaccharides being the major components that facilitate the Ag ion reduction and stabilize the surface of the Ag nanoparticle formed [10,22]. As observed in the UV–VIS, water-based AgNPs were concentrated, thus showing that more silver nanoparticles were synthesized as indicated by the spectrum in Figure 3 and Figure 4, image A. AgNPs were synthesized using water extracts in smaller sizes, yet they produced a higher yield.

#### 3.2.3. X-Ray Diffraction

**Figure 5:**
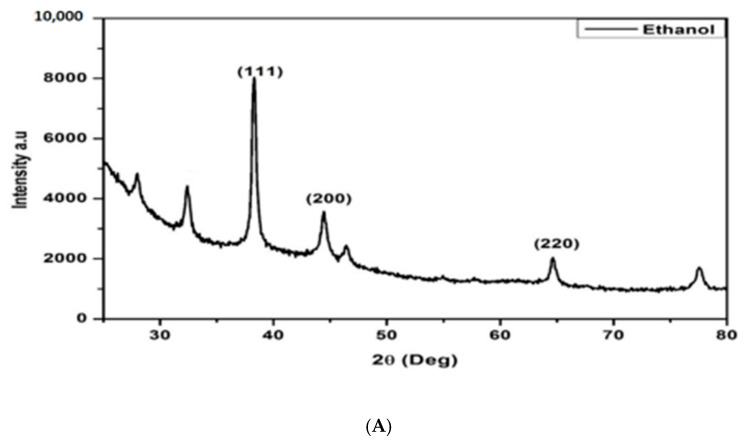

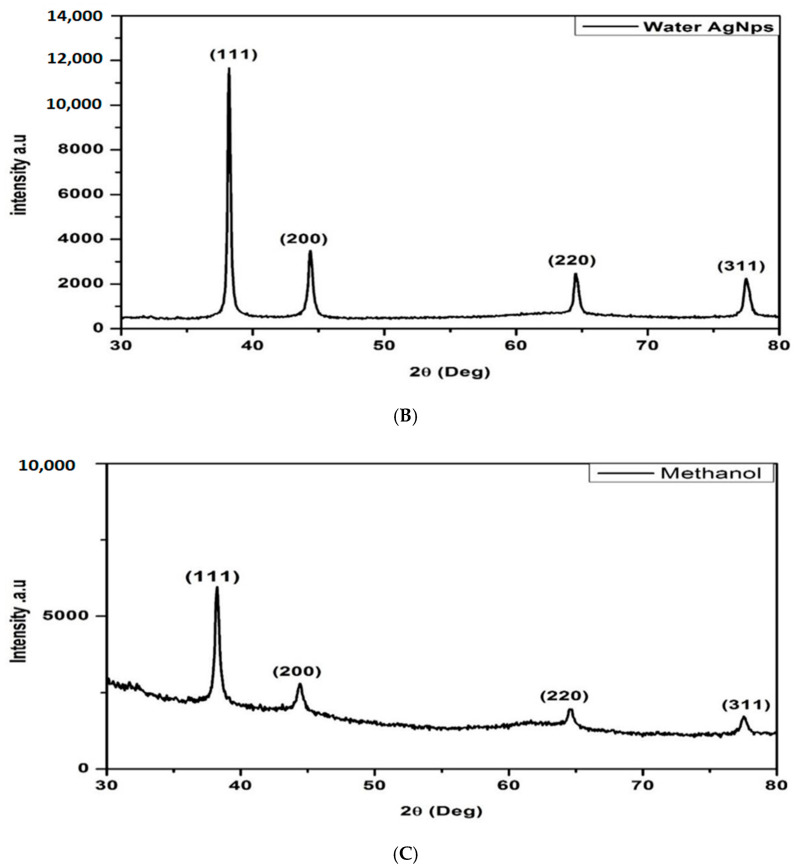
XRD diffractograms of silver nanoparticles formed using *E. elephantina* exudates extracted with ethanol (**A**), water (**B**), and methanol (**C**).

X-ray diffraction (XRD) is one of the most extensively used techniques for characterizing NPs. Typically, XRD provides information regarding the crystalline structure, nature of the phase, lattice parameters, and crystalline grain size [23]. The XRD patterns of the silver nanoparticles are shown in Figure 5. The diffracted intensities were recorded from 20° to 80°. Four strong Bragg reflections at 38.45°, 46.35°, 64.75°, and 78.05° correspond to the planes of (1 1 1), (2 0 0), (2 2 0), and (3 1 1), respectively, which can be indexed according to the facets of the face-centered cubic crystal structure of silver [23]. The interplanar spacing (d calculated) values are 2.336, 1.955, 1.436, and 1.224 Å for (1 1 1), (2 0 0), (2 2 0), and (3 1 1) planes, respectively, and matched with standard silver values. The average crystalline size was calculated using the Scherrer formula:

(1) $$D=\frac{k\lambda }{\beta \;cos\theta }$$

where *D* is the average crystalline size of the nanoparticles, *k* is the geometric factor (0.9), *λ* is the wavelength of the X-ray radiation source, and *β* is the angular FWHM (full-width at half maximum) of the XRD peak at the diffraction angle q [24]. The calculated average crystallite of the AgNP is ~25 nm for water, ethanol, or methanol.

### 3.3. Phytochemical Analysis

#### 3.3.1. Biochemical Analysis 

The traditional ways of preparing most plants for medicinal purposes involve boiling in water to yield a decoction or soaking plant material in freshly boiled water to produce an infusion [25]. In this study, boiling water was used to mimic, as closely as possible, the way traditional healers prepare *E. elephantina*; however, polar solvents (methanol and ethanol) were also used for extraction since they are known to be more active than lipophilic solvents [26].

Water as a solvent was able to extract more phytochemical compounds (Table 2) than ethanol and methanol solvents. It is interesting to note that the extracts of *E. elephantina* revealed the presence of phenolic, saponins, tannin, flavonoids, alkaloids, and terpenoids in ethanolic silver nanoparticles and methanolic nanoparticles, with the exception of alkaloids (Table 3). A similar study by McGaw et al. [27], using *Costus afer* leaves, showed the reason behind this phenomenon. It was proven that bioactive compounds of the plant extract were adsorbed over the spherically shaped nanoparticles, hence no detection of the initially present phytochemical compounds in the *E. elephantina*-capped silver nanoparticles. During the green synthesis, some phytochemicals might have undergone chemical transformation (oxidation, reduction, or hydrolysis), resulting in detectable forms of some compounds.

The crude extracts (Table 2) showed the presence of phenolics, tannins, terpenoids, and saponins. Saponins and tannins are well known antimicrobial and antioxidant active classes of bioactive phytochemicals in plants [28].

#### 3.3.2. Thin-Layer Chromatography

**Figure 6:**
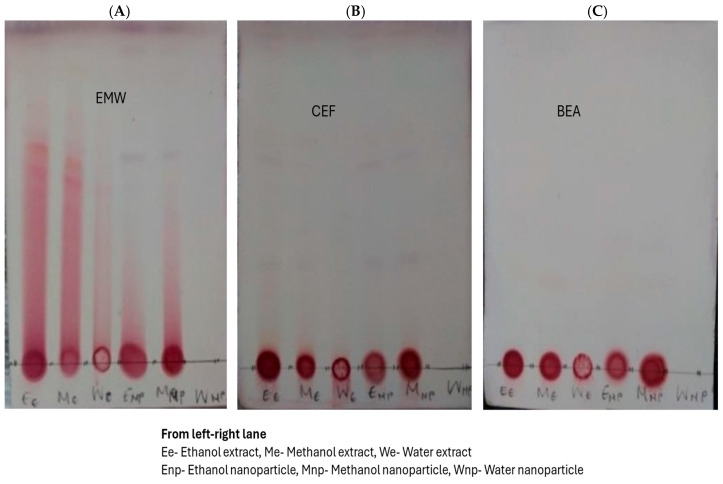
Photographs of TLC plates showing isolated extracts of *E. elephantina* tuber extracts and silver nanoparticles after treatment with the vanillin-sulfur solution, in three different solvents, (**A**) EMW, (**B**) CEF, and (**C**) BEA.

Thin-layer chromatography (TLC) is a commonly used qualitative analysis technique because it is cost effective, time efficient, and easy to perform [29]. TLC involves the use of different solvent systems that can dissolve compounds that move up the TLC plate, resulting in the development of chromatographs [30]. In this study, three solvent systems were used for TLC plates to separate the bioactive compounds present. The color, after applying the vanillin–sulfuric acid reagent, appears as blood-red (Figure 6). 

More fingerprint profiles were present in the ethyl acetate, methanol, water (EMW) chromatograph, as compared to–chloroform, ethyl acetate, formic acid (CEF), as well as in benzene, ethanol, ammonium hydroxide (BEA) chromatograph mobile phases. This indicates that the compounds are mostly polar. The intermediate solvent, CEF, had very few bands; however, the BEA developing system, which is non-polar, separated no compounds.

### 3.4. Antioxidant Testing

**Figure 7:**
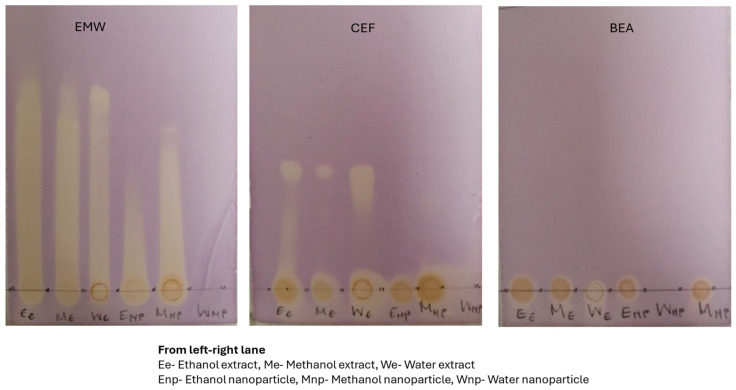
Photographs showing antioxidant activity of *E. elephantina* extracts and silver nanoparticles after treatment with DPPH.

The plant extracts from all three solvents had high antioxidant activities against the DPPH radicals as compared to the silver nanoparticles. The antioxidant activity analysis of the plant extracts on TLC were developed using three different solvent systems, BEA, CEF, and EMW, and sprayed with DPPH. The chromatograms revealed the presence of numerous different antioxidant compounds, which showed the characteristics of being polar to being more intermediate, when visualized using the DPPH spray reagent (Figure 7). The bands in Figure 7 show the positive scavenging activity of free radicals by methanol-extract-capped silver nanoparticles [M(AgNP)]. A study by Cowan [31] showed that these antioxidant properties are due to the simultaneous activity of phytochemical compounds, such as the phenols (compound present in the M (AgNP, as shown in Table 3)), which function as an antioxidant agent and AgNP as a catalyst for the process.

### 3.5. LCMS Analysis

Screening for phytochemical constituents in medicinal plants is of great value as it uncovers chemical substances with definite physiological action and gives scientific backing to their possible healing effects. LC/MS data showed the presence of a variety of chemical constituents with medicinal importance (Table 4).

### 3.6. AGAR Well Diffusion

In this study, agar well diffusion assay results clearly indicated that green synthesized silver nanoparticles of methanolic extracts have great anti-bacterial activity against *E. coli* (*ST38*), *K. pneumonia* (*ST307*) and *S. aureus* (*ST36*) (Thermo Fischer Scientific, Waltham, MA, USA). The methanol- and ethanol-extract-capped AgNPs had an increased antimicrobial activity against microorganisms compared to the crude extracts (Table 5). Water-extracts-capped silver nanoparticles had no activity against the bacteria; this could be due to the loss of phytochemicals (Table 4) that are required for antimicrobial activity, such as the flavonoids and terpenoids [36]. From the data in Table 5, it can be observed that silver nanoparticles enhance the activity of *E. elephantina* methanol and ethanol extracts.

Silver nanoparticles easily attach onto bacterial cell walls and penetrate the cells, due to their small sizes, which results in cell death, as the Ag+ from AgNP exhibits strong affinity to bind with the mercapto groups of bacterial proteins [37]. Such binding causes damage to bacterial DNA, thereby inhibiting bacterial replication, hence ultimately causing cell death [38].

Similarly, in a study by Ahmed and Ikram [39] these nanoparticles were synthesized via irradiation using an aqueous mixture of the *Ficus carica* leaf extract. *Cymbopogancitratus (DC) stapf* (commonly known as lemon grass), a native aromatic herb from India and cultivated in other tropical and subtropical countries, showed strong antibacterial effect against *P. aeruginosa*, *P. mirabilis*, *E. coli*, *Shigella flexneri*, *S. somenei*, and *K. pneumonia* [40]. Synthesized AgNPs have different sizes and surface chemistry properties and exhibit unique antimicrobial properties, allowing AgNPs to enter the bacteria and damage the cell walls [41]. 

Silver nanoparticles have been reported to have a wide range of applications, which are known for their antimicrobial properties, and have been used for years in the medical field for antimicrobial applications [11]. In recent years, many researchers have focused on the development of modified or novel synthetic strategies for silver nanoparticles in contrast to the use of conventional methods, which are strongly associated with toxic environmental footprints [12]. This study reports on the antimicrobial activities of silver-capped *E. elephantina* nanoparticles against pathogenic bacteria.

### 3.7. Cytotoxicity

**Figure 8:**
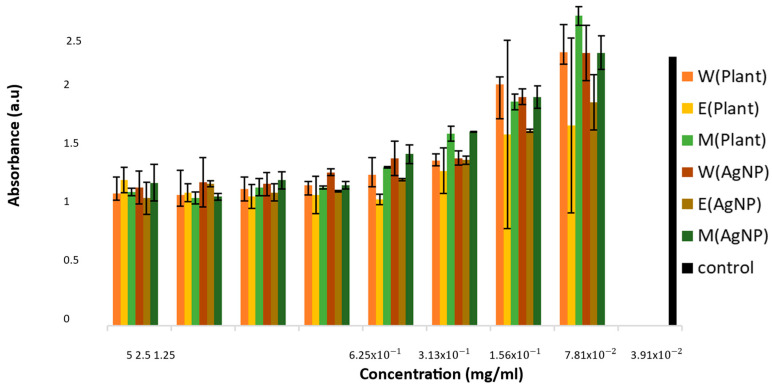
Optical density percentage of cytotoxicity test results from the MTS assay using HEK293 cells, treated with *E. elephantina* extracts and silver nanoparticles.

The MTS assay is mainly used to examine the viability and proliferation of cells and cytotoxicity. The principle is based on viable mammalian cells reducing the MTS tetrazolium compound to produce a colored formazan dye, which is soluble in cell culture media [42]. The formazan dye that was generated was then evaluated by measuring the absorbance at 490–500 nm [43]. The results of the MTS assay are presented in Figure 8. The cells treated with silver nanoparticles and *E. elephantina* extracts were compared with the negative control. After culturing in vitro, the optical density (OD) values for both silver nanoparticles and *E. elephantina* extracts increased coupling with a decrease in compound concentrations.

The in vitro study has shown the cytotoxicity profile of tested bare silver nanoparticles and crude extracts of *E. elephantina*. Silver nanoparticles had a slight toxic effect on the cells compared to the crude extracts from concentrations of 6.25 × 10^−1^ to 5 mg/mL, with the cell optical density (OD) ranging between 0.9077 and 2.2036 a.u for crude extracts and from 0.9681 to 1.9634 a.u for silver nanoparticles. Between concentrations of 3.13 × 10^−1^ mg/mL and 3.91 × 10^−2^ mg/mL, the nanoparticles showed less toxicity to the cells, with the OD ranging from 1.0134 to 1.9634 a.u and ranging from 0.9077 to 2.2036 a.u for crude extracts. The data showed that the silver nanoparticles improved the activity of *E. elephantina* extracts in reducing the toxic effect.

The methanol plant extracts [M(Plant)] and silver nanoparticles synthesized using methanol plant extract [M(AgNP)] compounds had a major increase in the OD compared to ethanol and water compounds. With methanol compounds containing a moderate number of phytochemical compounds and showing less toxicity to the cells, a study [44,45] demonstrated that phytochemicals play an important role in the viability of cells.

Table 4 shows that *E. elephantina* has abundant antioxidant agents that may influence cell viability. Zhang et al. [46] reported that the presence of antioxidants reduces toxicity towards cells. This was supported by a study performed by Nostro et al. [3], which reported that reactive oxidative species (ROS), such as superoxide and hydrogen peroxide, exhibited cytotoxic effects on spermatogonial stem cells (SSC). They showed that the addition of antioxidants to a preservation medium before freezing the cells increased their viability.

The AgNPs and plant extract were less toxic, promoting cell proliferation at lower concentrations and when the AgNPs had relatively small sizes. A study conducted by [47] indicated that the shape and size of nanoparticles may affect the viability of the cells. They further demonstrated that the increased cytotoxicity was a result of the increased process of aggregation of the small nanoparticles in the protein and ionic medium in a dosage-dependent approach. Nanoparticles that are about 10 nm in size tend to accumulate on the cell membrane or become clustered within the cell, leading to cell death due to the harmful effects of the aggregates [48,49].

## 4. Conclusions

This study illustrates the successful synthesis of silver nanoparticles (AgNPs) using *E elephantina* extracts, demonstrating their diverse morphologies and sizes, which are influenced by solvent variations. Methanol extracts yielded AgNPs with superior antimicrobial and cytotoxic activities, compared to other solvents. The rich phytochemical composition of *E. elephantina* greatly enhanced the antioxidant and antimicrobial properties of the AgNPs, making them effective against multidrug-resistant bacteria while, at the same time, exhibiting lower cytotoxicity towards human cells. Future research could focus on optimizing extraction methods to further improve nanoparticle efficacy, investigating the in vivo therapeutic potential, and exploring sustainable approaches for large-scale production. Harnessing plant-based NPs holds promise for developing advanced antimicrobial agents with minimal cytotoxicity, paving the way for innovative biomedical applications.

This study revealed that the extracts of *Elephantorrhiza elephantina* are a good basis for synthesis of silver nanoparticles. The reduction of silver ions by the plant’s extracts resulted in the formation of stable particles with nano sizes. The formation was confirmed by the color changing to brown, after 24 h. The microscopic images and spectroscopic characterizations using UV–VIS, TEM, and XRD were useful in proving the formation of particles and in confirming their size, shape, and morphology. The shapes were spherical, triangular, and irregular because of the solvent type used; sizes were fairly small, thus allowing for better penetration through the cell membrane and into pathogenic cells. The antibacterial efficacy against some bacteria confirmed that the AgNPs are capable of producing antibacterial efficacy and strengthening the medicinal value of plants. The MTS assay showed that the silver nanoparticles were less toxic towards the HEK293 cells at lower concentrations of the silver nanoparticles and plant extracts. The sizes and shapes of nanoparticles, therefore, play a role in the cytotoxicity and antimicrobial effects.

The exact compounds that are responsible for antimicrobial and free-radical-scavenging activities and compounds that reduce the silver nitrate to form silver nanoparticles are not yet known. The separation of compounds that are found in crude extracts by making use of column chromatography, thus, can be important as different fractions that can be evaluated for biological activities. The separation will be essential in studying the various compounds that *E. elephantina* and silver-capped nanoparticles possess.

## Figures and Tables

**Table 1:** Parameters for cytotoxicity studies with the MTS assay as determined with trypan blue exclusion.

Cell Line	Repetitive Experiment	Exposure Time	Cell Number cells/mL	Viability (%)	Incubation (h)
HEK293	I	72	1.54 × 10	≥94	2 & 4
HEK293	II	72	3.66 × 10	≥54	2 & 4
HEK293	III	72	9.48 × 10	≥78	2 & 4

**Table 2:** Phytochemical constituent analysis of *E. elephantina* extracts. (+ indicates the presence of the phytochemical compounds. − indicates the absence of the compounds).

	Distilled Water	Ethanol	Methanol
Terpenoids	+	+	+
Tannins/Phenolics	+	+	+
Alkaloids	−	−	−
Saponins	+	−	−
Flavonoids	−	−	−

**Table 3:** Phytochemical constituent analysis of green synthesized silver nanoparticles. (+ indicates the presence of the phytochemical compounds. − indicates the absence of the compounds).

	Distilled Water	Ethanol	Methanol
Terpenoids	−	+	+
Tannins/Phenolics	−	+	+
Alkaloids	−	+	−
Saponins	+	+	+
Flavonoids	−	+	+

**Table 4:** Phytochemical compounds extracted using water, that were detected in *E. elephantina*, showing significant biological activities.

Compound Name	Phytochemical Class	Biological Activity	References
Ellagic acid	Polyphenol	Antioxidant Anti-cancer (cytotoxic to cancer cells) Wound healing Liver problems Anti-proliferative	[32]
Procyanidin B1	Polyphenol flavonoid	Inhibits hepatitis c virus RNA replication Exhibit antiinflammatory effects	[33]
Quercetin	Flavonoid	Anti-viral Anti-inflammatory Antioxidant	[2]
1,3,6-tri-Ogalloylglucose	Phenols	Anti-inflammatory	[34]
Myricetin	Flavonoid	Antioxidant Antineoplastic agent/cytotoxic	[35]
Robinetin	Flavonol	Antioxidant	https://pubchem.ncbi.nlm.nih.gov/compound/Robinetin#:~:text=Robinetin%20a%20pentahydroflavone%20that,ChEBI Access date: 2 February 2025

**Table 5:** Antimicrobial activity of *E. elephantina* extracts and green synthesized silver nanoparticles’ inhibition zones measured in millimeters (mm).

Microorganisms	*E. elephantina* Extracts			Silver Nanoparticles		
	Methanol	Ethanol	Water	Methanol	Ethanol	Water
*E. coli*	6	0	12	10	6	0
*S. aureus*	7	0	12	12	6	0
*K. pneumoniae*	0	0	8	14	9	0

## Data Availability

We have included all data related to this study in the main text.
